# Spectrum of offending drugs and cutaneous adverse drug reactions requiring hospitalisation in a tertiary South African hospital in TB/HIV endemic setting

**DOI:** 10.3389/falgy.2024.1481281

**Published:** 2024-11-28

**Authors:** S. P. P. Konyana, N. F. Teixeira, L. Pirjol, B. Thwala, W. Nkoyane, M. Porter, F. Gxolo, E. Phillips, R. Lehloenya, A. Mankahla, J. Peter

**Affiliations:** ^1^ Division of Dermatology, Department of Medicine, Nelson Mandela Academic Hospital, Walter Sisulu University, Mthatha, South Africa; ^2^ Division of Allergy and Immunology, Department of Medicine, Groote Schuur Hospital, University of Cape Town, Cape Town, South Africa; ^3^ Division of Dermatology, Department of Medicine, Groote Schuur Hospital, University of Cape Town, Cape Town, South Africa; ^4^ Center for Drug Safety and Immunology, Department of Medicine, Vanderbilt University Medical Center, Nashville, TN, United States; ^5^ Institute for Immunology and Infectious Diseases, Murdoch University, Murdoch, WA, Australia

**Keywords:** drug reaction, CADR, SCAR, PWH, SJS/TEN, dress, co-trimoxazole, art

## Abstract

**Introduction:**

Cutaneous immune-mediated adverse drug reactions are more prevalent in people with human immunodeficiency virus (PWH). Severe cutaneous adverse drug reactions (SCAR) are a life-threatening subset of cutaneous adverse drug reactions (CADRs) and a significant public health issue in settings endemic for human immunodeficiency virus and tuberculosis. However, limited data are available on CADR requiring hospitalisation in African settings. The aim of this study is to describe the epidemiology, offending drugs and outcomes of CADRs requiring admission to a South African tertiary dermatology service.

**Methods:**

Retrospective folder review was conducted on all CADRs requiring hospitalisation at Nelson Mandela Academic Hospital in Mthatha, Eastern Cape, South Africa between 30 July 2015 and 15 December 2022. This data was compared to prospective inclusion of CADR admissions between 03 March 2021 and 09 April 2024 as part of the Immune-Mediated Adverse Drug Reactions (IMARI) Registry and Biorepository and AFRISCAR consortium. Where possible, phenotype and drug causality assessment was performed through RegiSCAR, or Naranjo and/or ALDEN scoring respectively.

**Results:**

CADR admissions included 122 cases: 89 and 33 in the retrospective and prospective cohorts respectively. The commonest SCAR phenotype was Stevens-Johnson syndrome/toxic epidermal necrolysis (SJS/TEN) at 59.8% (73/122), although other validated SCAR phenotypes included drug reaction with eosinophilia and systemic symptoms (DRESS), acute generalized exanthematous pustulosis (AGEP) and generalized fixed bullous drug eruption (GBFDE). Cutaneous presentations included typical and atypical SCAR features against a background Fitzpatrick skin tones of type IV and above. Amongst the retrospective cohort 16.9% (15/89) of phenotypes were unclassifiable due to lack of photographs. The overall median (IQR) age was 38 (25–50) years, 50.8% (62/122) were male and 60.7% (74/122) were PWH [median (IQR) CD4T-cell count of 267 (76–470) cells/mm^3^]. The commonest offending drugs included cotrimoxazole in 24.6% (30/122); and anti-retroviral therapy (ART) in 13.9% (17/122). No offending drug could be identified in 24.7% (22/89) of the retrospective cohort. The median (IQR) length of hospital stay for validated SCAR was 13 (8–21) days for the retrospective cohort and 19 (13–28) days for the prospective cohort (*p* = 0.03). The median (IQR) length of hospital stay for non-SCAR was 9 (5–13) days for the retrospective cohort and 11 (9–16) days for the prospective cohort.

**Conclusion:**

Typical and atypical presentations of SCAR were represented in this vulnerable South African cohort of predominantly PWH. SJS/TEN was the commonest phenotype, and cotrimoxazole the most frequent offending drug. This data emphasises the need for prospective data collection across a diverse African population for valid SCAR phenotyping and drug causality assessment.

## Introduction

Cutaneous adverse drug reactions (CADRs) are predominantly delayed immune-mediated reactions. In human immunodeficiency virus (HIV) and tuberculosis (TB) endemic settings they occur most frequently amongst people with HIV (PWH), with offending drugs reflecting prescribing practices of antiretrovirals and antibiotics (1). CADRs are significantly more common in PWH and TB with an incidence reported of up to 27% (2). Severe cutaneous adverse drug reactions (SCARs) are a subset of CADRs that pose a significant public health burden in these vulnerable populations, largely centred in African lower- and middle-income countries.

There are four types of SCAR, including drug reaction with eosinophilia and systemic symptoms (DRESS), Stevens-Johnson syndrome/toxic epidermal necrolysis (SJS/TEN), acute generalized exanthematous pustulosis (AGEP), and generalized fixed bullous drug eruption (GFBDE). Less severe CADRs include morbilliform drug eruption (MDE), lichenoid drug eruption (LDE) and fixed drug eruptions (3, 4).

SJS and TEN are differentiated only by percentage of body surface area (BSA) affected. SJS affects less than 10% of the BSA, TEN affects more than 30% of the BSA and SJS/TEN overlap affects a range of 10%–30% of the BSA (4). The clinical features of SJS and TEN are epidermal necrolysis, skin detachment and blistering of the mucosal membranes (5). The mortality rate of SJS ranges from 1% to 4% and that of TEN can vary from 12% to 50% depending upon the severity-of- illness score for toxic epidermal necrolysis (SCORTEN) (6). Drugs that commonly cause SJS and TEN include sulphonamides, anti-epileptics, non steroidal anti-inflammatory drugs (NSAIDS), allopurinol, and nevirapine (an antiretroviral drug). Drugs frequently associated with DRESS include anti-epileptics, sulphonamides, allopurinol, nevirapine and abacavir (also an antiretroviral) (7).

DRESS is characterized by eosinophilia, facial oedema, erythroderma, pyrexia, lymphadenopathy, atypical lymphocytes, and internal organ (such as liver and renal) involvement (5). DRESS presents 3 weeks to 3 months following treatment initiation with the causative drug and its mortality rate ranges from 10% to 20% (8).

All types of SCAR are concerning due to their high mortality rates (up to 50%) (2), treatment-interruption related effects upon infectious diseases, SCAR related long-term morbidity (9) and health care costs due to prolonged hospitalisation (2). Despite this disease burden, there remains a paucity of published literature from such settings, particularly from African countries. Furthermore, there is underrepresentation of African populations and detailing of cutaneous presentations of CADRs amongst darker Fitzpatrick skin tones, with potential consequence for early clinical recognition and outcomes (10). This first aim of this study is thus to report on and describe the burden of disease from CADRs in African TB/HIV endemic settings.

The European Registry of Severe Cutaneous Adverse Reactions to Drugs (RegiSCAR) has established a validation system to standardize the classification of SCAR (3). Cases are reviewed by dermatologists using a validation form and scoring system that considers clinical information, photographs, and laboratory results (including blood tests and histology) to assign one of the four validated phenotypes. Similarly, Drug causality assessment tools are available for determining the likelihood of drug causality in CADRs (4, 11–16). These include the Naranjo and ALDEN drug causality assessments are the most well-known, with Naranjo used for DRESS, AGEP, and GFBDE, and ALDEN specific to SJS/TEN (11, 12). Thus, the second aim of this work was to assess how effective routine clinical records in a tertiary dermatology service could be for SCAR validation and drug causality assessment, and hence whether the resources required for prospective case recruitment, validations and drug causality assessments were justifiable.

## Materials and methods

The study design was an observational cohort study, with two arms: retrospective and prospective. Data was collected from records of patients admitted to Nelson Mandela Academic Hospital (NMAH), Mthatha, Eastern Cape Province, South Africa from 30 July 2015 to 15 December 2022 with a clinical diagnosis of CADR. To find eligible patients for retrospective study enrolment we examined NMAH dermatology ward admission registries. For data collection we accessed information from multiple sources including: patient folders, the hospital and provincial death registries and National Health Laboratory Services databases.

Data collected included demographics, drug history, CADRs phenotype information, comorbidities, length of hospital stay and cause of mortality. The prospective study data was from the Immune-Mediated Adverse Drug Reactions (IMARI) Registry and Biorepository initiated at the University of Cape Town and part of the AFRISCAR consortium initiative to enrol SCAR cases across diverse African populations. The registry collects clinical, laboratory as well as biological samples of cases suspected to have had a treatment-limiting and/or life-threatening CADR (17). It also allows for the storage of images of patients enrolled to ensure accurate validations can occur retrospectively once all necessary data is captured and reviewed. The data analysed in this cohort was from patients enrolled in the database starting from March 2021 to April 2024. During this period, there were two inpatient death records of SJS/TEN found for patients that were not enrolled in the prospective IMARI database, and this data was analysed as part of the retrospective cohort given limited mortality information. Patients who were below the age of 12, patients with drug reactions that did not meet the definition of CADR and patients with missing hospital records were excluded from the study. The retrospective and prospective components of the study were approved by the Human research Ethics committees (HREC), including the use of photographs, at both Walter Sisulu University (WSU) (HREC: 056/2020) and University of Cape Town (HREC R031/2018).

Qualified dermatologists performed phenotype validations for the prospective arm, whilst case validations for all retrospective cases were performed by two dermatologists based at WSU. The RegiSCAR validation scoring system classified SCAR cases as: definite, probable, possible, and not a case of SCAR. Drug causality assessments used to identify the offending drugs in SJS/TEN cases was the ALDEN Score, while the Naranjo drug assessment tool was used to assess drug causality in all other phenotypes of CADR (11, 12). The SCORTEN is an illness severity-scoring system validated for SJS-TEN, which uses seven clinical parameters, allotted equal weight to predict probability of mortality (6). However, it was not part of the tools used in this study.

Data analysis was performed using Excel and GraphPad Prism version 10.3.0. Data analysis predominantly involved descriptive analyses. Where required, observed and expected frequencies for each variable in both retrospective and prospective cases were compared using a chi-squared test. Continuous variables such as age and length of hospital stay were compared with Mann-Whitney *U*-test for non- parametric data.

## Results

### Cutaneous manifestations of SCAR in study population

**Figure 1:**
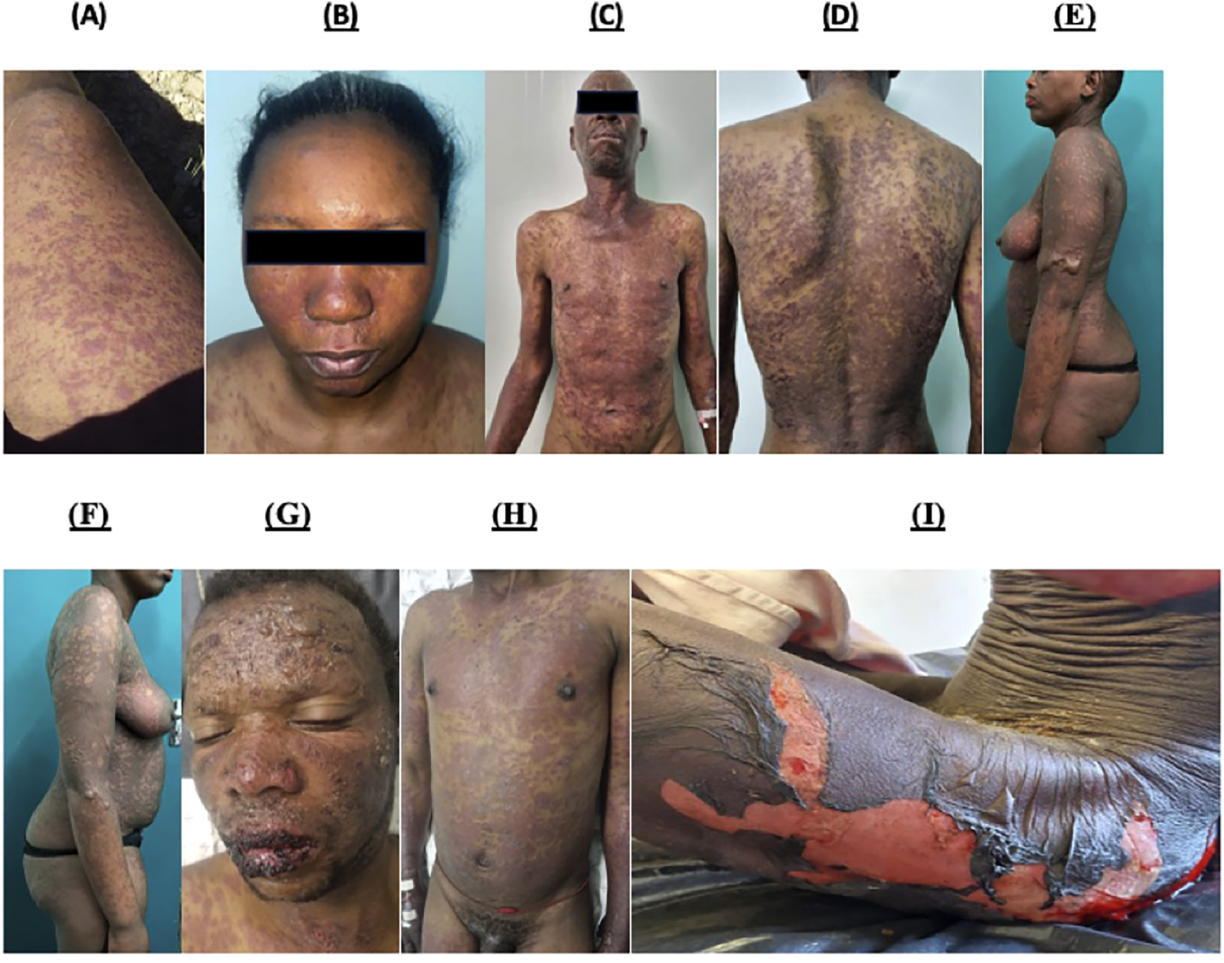
Photographs of validated cases obtained from the prospective IMARI database showing typical and atypical cutaneous features of SCAR presentations in higher Fitzpatrick skin tones. **(A)** Probable DRESS, Day 4 from the onset of symptoms. Erythematous and purpuric papules, coalesced plaques with mild induration involving the right thigh and knee. **(B)** Probable DRESS, Day 9 from the onset of symptoms. Facial oedema with erythematous morbilliform eruption with centro-facial and upper chest involvement. **(C)** and **(D)** Definite SJS/TEN, Day 7 from onset of symptoms. Extensive, erythematous and purpuric papules and plaques with atypical targetoid lesions affecting the neck, trunk and upper limbs. **(E)** and **(F)** Definite SJS/TEN, Day 8 from onset of CADR. Extensive dusky colored patches with flaccid bullae more pronounced on the Left arm than the right arm and the lower abdominal area. Features of epidermal necrolysis affect the lower half of the face and neck. **(G)** and **(H)** Definite SJS/TEN, Day 10 from onset of CADR. Extensive facial bullae with haemorrhagic crusting with sparing of the eyes. Extensive dusky coloured and purpuric patches and plaques with targetoid lesions on the-trunk and upper limbs. **(I)** Definite SJS/TEN, Day 10 from onset of symptoms. Marked areas of denuded skin on the trunk, thighs and legs, with skin stripping, Nikolsky sign and bullae in surrounding areas.

Figures 1A–F shows a selection of photographs from the prospective IMARI cohort highlighting key cutaneous features. Pictures highlight the appearance of erythematous and purpuric papules and atypical targetoid features during the progression to more severe epidermal necrolysis.

### Demographics, co-morbidities, CADR phenotypes and outcomes of admissions

**Figure 2:**
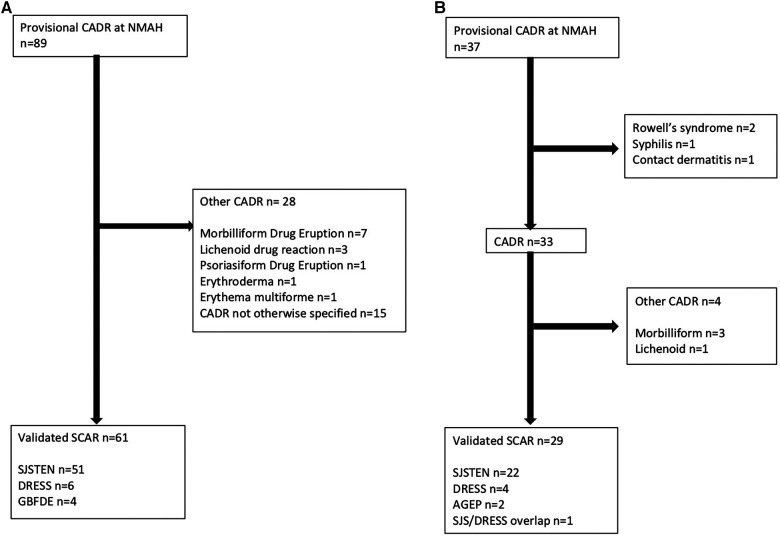
**(A)** Consort diagram of retrospective provisional CARDs at NMAH between 30 July 2015 and 15 December 2022. **(B)** Consort diagram of prospective provisional CARDs at NMAH between 03 March 2021 and 09 April 2024.

A total of 122 cases were admitted with CADR to the tertiary dermatological services at NMAH during the study period (30 July 2015–09 April 2024). Figure 2 shows the consort diagram of included CADRs stratified by retrospective (*n* = 89) (A) and prospective (*n* = 33) (B). As seen in Table 1, in the retrospective arm, 49.4% (44/89) were female with a median age of 36 (IQR 26–50) years, and 55.1% (49/89) were PWH (Table 1). A validated SCAR was confirmed in 68.5% (61/89), with the commonest phenotype of SJS/TEN [83.6% (51/61)] (Figure 2A). In 16.9% (15/89) there was insufficient information from folder review to determine CADR phenotype (Table 1). In the prospective arm, 37 cases were identified as having a provisional CADR at NMAH from March 2021 to April 2024, of which 33 were confirmed as CADR. Four of the CADR were categorized as “other”, which included three MDE and one LDE. The total number of validated SCAR cases was 29 (Figure 2B), with SJS/TEN being the commonest at 66.7% (22/33), followed by DRESS at 12.1% (4/33) and thirdly AGEP at 6.1% (2/33) (Figure 2B). As seen in Table 1, CADR cases had a median age of 41 (IQR 36–49) years; 48.5% (16/33) were female; and 75.8% (25/33) were PWH, of which 44.8% (13/29) were on antiretroviral treatment (ART) at the time of the initiation of the CADR and 41.4% (12/29) were on cotrimoxazole prophylaxis (Table 2). Current TB was present in 41.4% (12/33) of cases (Table 1). All of the validated prospective CADR except one had pulmonary TB (11/12); all TB cases were all receiving first-line anti-TB treatment (Table 2).

**Table 1:** Demographics, co-morbidities, CADR phenotypes and outcomes of admissions between 30 July 2015 and 09 march 2024, stratified by retrospective or prospective inclusion.

		Overall *n* = 122 (%)	Retrospective *n* = 89 (%)	Prospective *n* = 33 (%)	*p*-value
Female		60 (49.2)	44 (49.4)	16 (48.5)	0.947
Median age (years) (IQR)		38 (28–50)	36 (26–50)	41 (36–49)	0.093
Phenotype	SJS/TEN	73 (59.8)	51 (57.3)	22 (66.7)	0.553
	DRESS	10 (8.2)	6 (6.7)	4 (12.1)	0.355
	GBFDE	4 (3.3)	4 (4.5)	0 (0.0)	0.224
	Morbilliform drug eruption	10 (8.2)	7 (7.9)	3 (9.1)	0.831
	Missing phenotype	15 (12.3)	15 (16.9)	0 (0.0)	**0** **.** **018**
	Other^a^	10 (8.2)	6 (6.7)	4 (12.1)	0.355
Comorbidities^b^	HIV	74 (60.7)	49 (55.1)	25 (75.8)	0.193
	TB	32 (26.2)	18 (20.2)	14 (42.4)	**0** **.** **034**
	Epilepsy	19 (15.6)	14 (15.7)	5 (15.2)	0.943
	Hypertension	13 (10.7)	7 (7.9)	6 (18.2)	0.121
	Other^c^	17 (13.9)	13 (14.6)	4 (12.1)	0.383
	Missing comorbidity information	7 (5.7)	7 (7.9)	0 (0.0)	0.107
Median length hospital stay (days) (IQR)	SCAR cases	13 (10–21)	13 (8–21)	19 (13–28)	**0** **.** **029**
Mortality^d^		16 (13.1)	12 (13.5)	4 (12.1)	0.854

^a^ Other phenotype: Retrospective: *n* = 1 Erythroderma, 3 Lichenoid drug eruption, 1 Erythema multiforme, 1 Psoriasiform drug eruption. Prospective: *n* = 2 AGEP, 1 SJSTEN/DRESS overlap, 1 Lichenoid drug eruption.

^b^ Some patients have >1 comorbidity.

^c^ Other comorbidities: Retrospective: 2 Diabetes, 2 Schizophrenia, 1 Anaemia, 1 Chronic kidney disease, 1 Dystonia, 1 Hypokalaemia, 1 Myeloid leukaemia, 1 Rheumatoid arthritis, 1 Seborrheic dermatitis, 1 Pleural effusion, 1 Cerebrovascular accident Prospective: 1 Diabetes, 1 Gout, 1 Valvular heart disease, 1 Major depressive disorder.

^d^ In-patient deaths: Prospective: *n* = 4 deaths, of which 3 SCAR related. Retrospective: *n* = 12 retrospective deaths, of which 1 SCAR related, 3 non-scar related, and 8 unknown cause of death.

Bold values represent statistically significant *p* values.

**Table 2:** HIV, TB and medications of RegiSCAR validated SCAR in prospective arm, stratified by RegiSCAR category.

	Total cases *n* = 29^a^ (%)	Total SJS-TEN *n* = 22 (%)	SJS-TEN *n* = 22 (%)			Total DRESS *n* = 4 (%)	DRESS *n* = 4 (%)		Total AGEP *n* = 2 (%)
			Definite (*n* = 16)	Probable (*n* = 4)	Possible (*n* = 2)		Probable (*n* = 2)	Possible (*n* = 2)	
Female	13 (44.8)	9 (40.9)	6 (37.5)	2 (50.0)	1 (50.0)	2 (50.0)	2 (100.0)	2 (100.0)	2 (100.0)
PLHIV	22 (75.9)	17 (77.3)	12 (75.0)	4 (100.0)	1 (50.0)	3 (75.0)	2 (100.0)	1 (50.0)	2 (100.0)
On ART	13 (44.8)	10 (45.5)	7 (43.8)	2 (50.0)	1 (50.0)	1 (25.0)	1 (50.0)	0 (0.0)	2 (100.0)
Median CD4 (IQR)	258 (80–395)	206 (80–395)	161 (66–400)	268 (197–334)	168 (168–168)	316 (182 −330)	330 (323–336)	47 (47–47)	528 (528–528)
Co-trimoxazole prophylaxis	12 (41.4)	9 (40.9)	7 (43.8)	2 (50.0)	0 (0.0)	3 (75.0)	2 (100.0)	1 (50.0)	0 (0.0)
TB	12 (41.4)	10 (45.5)	8 (50.0)	2 (50.0)	0 (0.0)	2 (50.0)	1 (50.0)	1 (50.0)	0 (0.0)
Pulmonary TB	11 (37.9)	9 (40.9)	7 (43.8)	2 (50.0)	–	2 (50.0)	1 (50.0)	1 (50.0)	–

^a^ 1 case, male, SJS/TEN and DRESS overlap, HIV negative, TB negative. This case was not included in the individual SJS/TEN, DRESS cases.

The leading comorbidity (Table 1) in both retrospective and prospective cohorts was HIV, the total PWH being 55.1% (49/89) and 75.8% (25/33) respectively (*p* = 0.19). The second most common comorbidity was TB, seen in 20.2% (18/89) of retrospective cases and 42.4% (14/33) of prospective cases (*p* = 0.03). Epilepsy was the next commonest co-morbidity affecting 15.7% (14/89) and 15.2% (5/33) of the retrospective and prospective cohorts respectively. 14.3% (2/14) of the retrospective patients with epilepsy had anti-epileptics as their offending drug, while all five of the prospective cases with epilepsy had anti-epileptics as their offending drug. Notably, the offending drug information for 28.6% (4/14) of the retrospective epileptic patients was missing. Other comorbidities recorded included hypertension (7.9% in retrospective cases and 18.2% in prospective cases), diabetes and schizophrenia.

The median duration of hospital stay in SCAR cases was 13 (IQR 10–21) days compared to non-SCAR cases of 9 (IQR 6–13) days. Median length of hospital stay was longer for SCAR cases in the prospective vs. retrospective cohorts [19 (13–28) vs. 13 (8–21) days, *p* = 0.03] (Table 1). Of the 122 admitted CADR cases, inpatient fatal outcomes were observed in 16 cases accounting for a 13.1% mortality rate. Of the four deaths noted in the prospective data, three of these were SCAR-related (75.0%), and all three validated as a definite SJS/TEN. Of the 12 retrospective deaths, all had SJS/TEN, one of which was noted to be SCAR-related and three were non-SCAR related. The remaining eight deaths (66.7%) in the retrospective cohort had missing/insufficient data to determine the cause of death.

### Offending drug(s) causing the CADR

**Figure 3:**
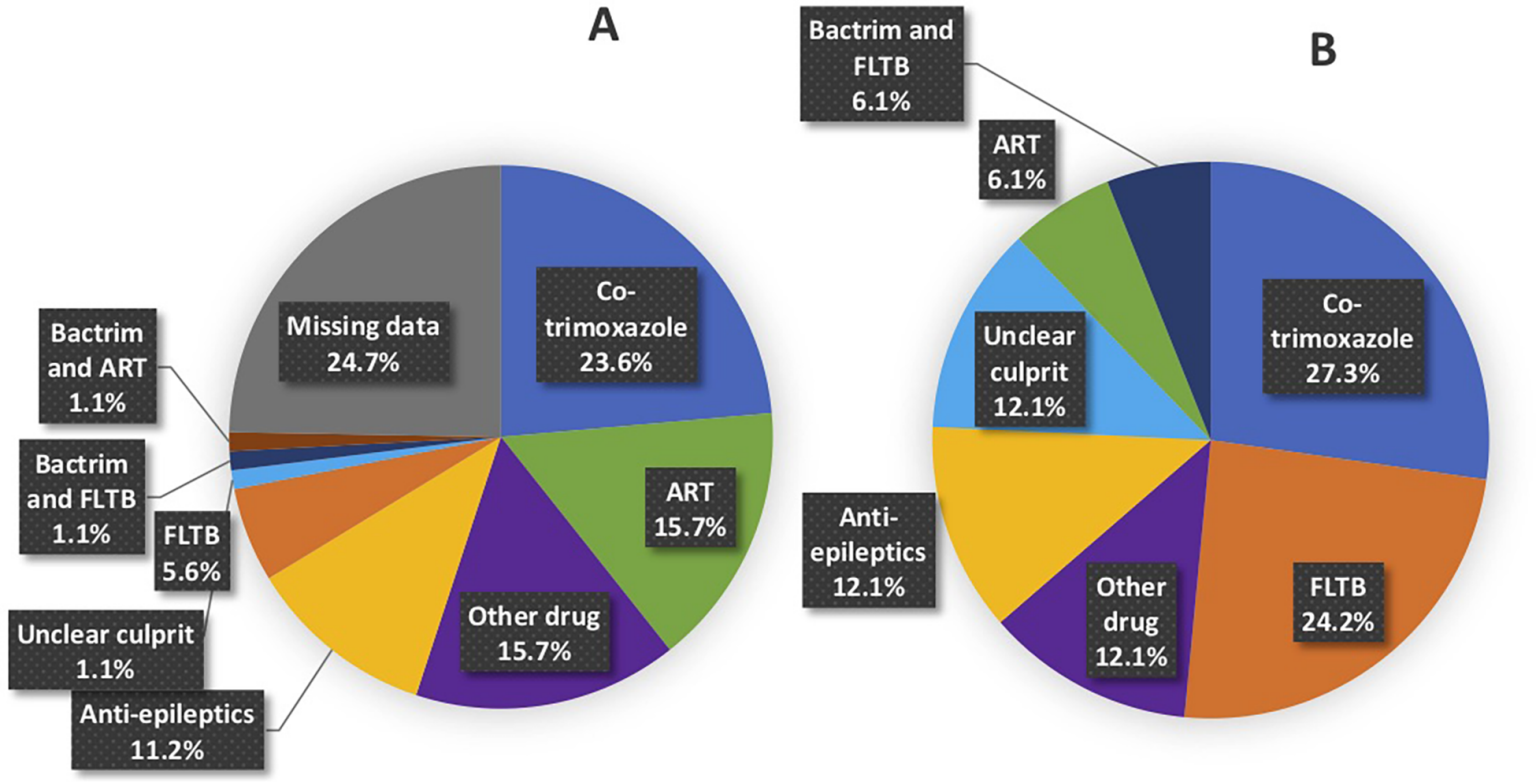
Breakdown of offending drug(s) based on drug causality assessment tools in **(A)** retrospective and **(B)** prospective cases. Breakdown of anti-epileptic drugs. **(A)** Retrospective: 7 (7.9%) phenytoin, 2 (2.2%) lamotrigine and 1 (1.1%) carbamazepine. **(B)** Prospective: 3 (9.1%) lamotrigine, 1 (3.0%) phenytoin.

Figures 3A,B are pie charts of the offending drugs identified by conducting drug causality assessments. Offending drugs with the highest drug causality assessments in the 89 retrospective CADRs included cotrimoxazole in 23.6% (21/89), Antiretrovirals (ARTs) in 15.7% (14/89), anti-epileptics in 11.2% (10/89) and first line ant-tuberculosis drugs (FLTBDs) medication in 5.6% (5/89). A total of 24.7% (22/89) of the retrospective cases had insufficient information to identify the casual drug due to missing data. In contrast, of the 33 prospective all drug causality assessments could be competed. Cotrimoxazole and anti-epileptics were implicated in similar frequencies to the retrospective cohort, but in the prospective cohort, a greater number of cases were linked to FLTBD treatments, as well as the recognition of other known CADR offending drugs such as allopurinol and NSAIDs.

## Discussion

**Figure 4:**
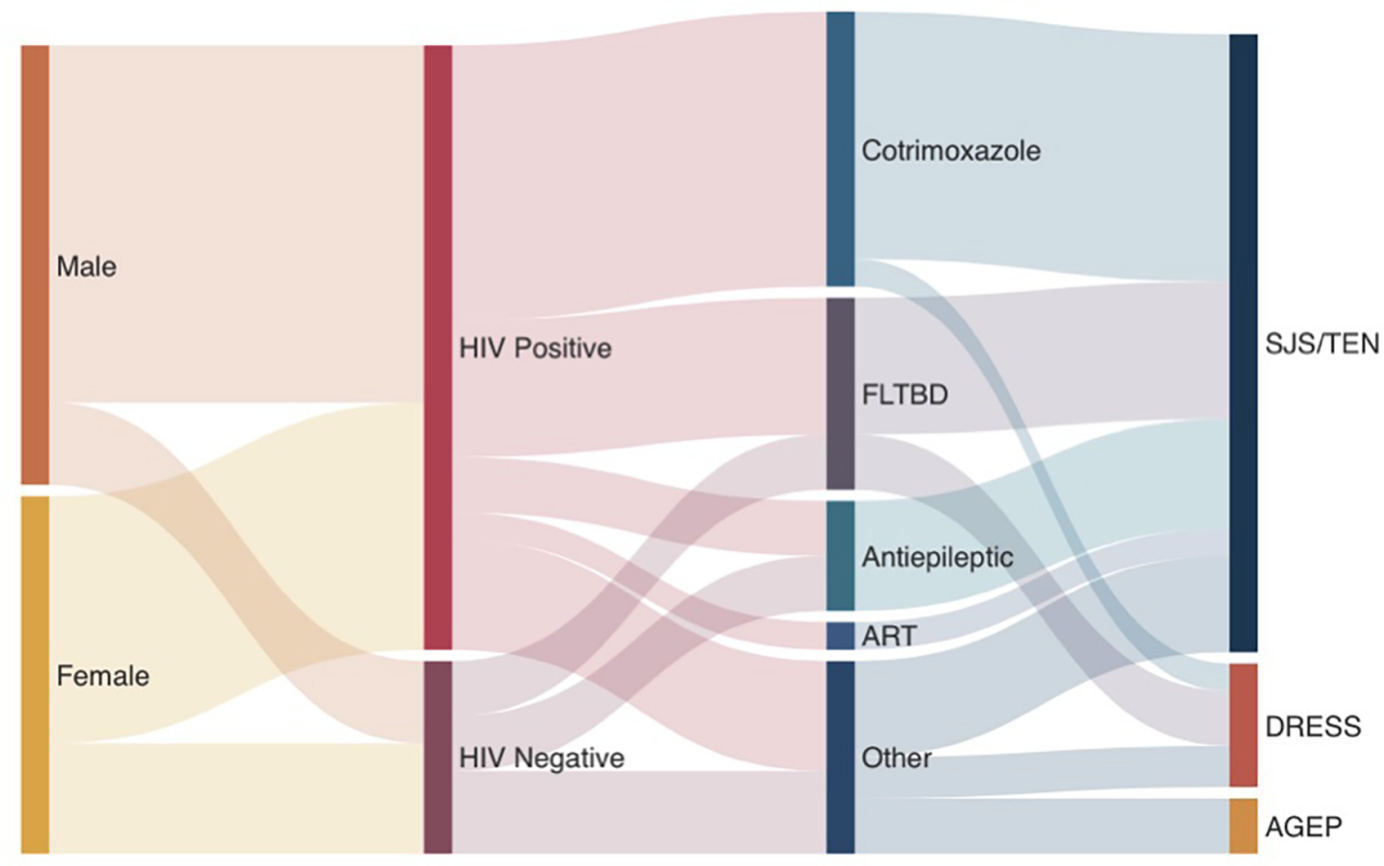
Sankey diagram of RegiSCAR validated SCAR cases in prospective arm. “Other” treatments include miracell, allopurinol, NSAIDs, Adcodol and unknown causative agent.

SCARs are a group of life-threatening, treatment-limiting delayed hypersensitivities with mortality rates of up to 50% and considerable long-term morbidity; several have strong class I HLA genetic associations (7). This includes the HLA-B* 13:01 allele associated with cotrimoxazole and dapsone-associated DRESS (18–20), and analysis into these genetic associations is ongoing in this cohort. Early recognition, identification and cessation of the offending drug(s), and multidisciplinary supportive inpatient care are critical for good outcomes and reduced long-term sequelae. In TB and HIV endemic settings, SCARs disproportionately impact vulnerable PWH with advanced immunosuppression, and frequent needs for prolonged multi-drug antibiotic regimens for co-infections like TB (21). Unfortunately, to date there has been a paucity of SCAR, or CADR data from diverse African populations that highlight the range of cutaneous presentations, common offending drugs and outcomes. Given this background, the key findings of this cohort of CADRs requiring admission to a tertiary dermatology unit in Mthatha, South Africa include: (i) over 60% of SCAR occurs in PWH and advance immunosuppression, (ii) SJS/TEN is the commonest SCAR phenotype, although all other major SCARs were diagnosed at lower prevalence, potentially due to a bias in less severe phenotypes not presenting to hospital (iii) cutaneous features included typical and atypical features SJS/TEN and DRESS features in a population with darker skin tones, (iv) cotrimoxazole and antiretrovirals are the commonest offending agents (Figure 4), and (v) up to a quarter of phenotyping and drug causality assessments were not possible due to a lack of pictures of drug exposure details in the retrospective study of admission CADRs.

PWH are estimated to have an up to 100-fold increased risk of SCAR, although precise estimates of prevalence and the permissive mechanisms of HIV-associated immune dysregulation are poorly defined (22). The decline in CD4T-cell count leads to an increasing risk of opportunistic infections, such as Pneumocystitis jiroveci pneumonia that require prophylaxis with cotrimoxazole, and exponential rise in the development of active TB (23). In line with this, the commonest culprit drugs causing CADR and SCAR in PWH are cotrimoxazole and ART and this cohort is younger compared to typical SCAR cohorts from non-HIV endemic settings (24). This is a similar spectrum of culprit drugs to that reported in our Cape Town based South African cohort (25). Notably, the second commonest causative drug in the retrospective cohort was ART, more specifically efavirenz and nevirapine. This could be due to its previous use as first line therapy during the time period in which retrospective data was analysed. However, in contrast to the Cape Town SCAR cohort where DRESS outnumbers SJS/TEN 3:1 (25), SJS/TEN is the predominant SCAR phenotype in Mthatha, accounting for nearly two thirds of all validated cases – both in the retrospective and prospective study arms. Given the TB prevalence is similar in Eastern and Western Cape (26), it is unclear if this represents a referral bias – perhaps with less severe CADRs of the DRESS spectrum undiagnosed and managed in peripheral hospitals, or a different population genetic background e.g., HLA allele frequencies between higher rates of mixed ethnicities in the Western vs. Eastern Provinces (27–29). Outside of the burden of disease in PWH, the full spectrum of CADR and SCARs were noted in our cohort, with known co-morbidities and drug culprits such as antiepileptics, NSAIDs and allopurinol (30). Although SCARs can affect any age group, the incidence varies with age, generally, older adults are at higher risk due to polypharmacy and physiological changes (31). These changes may be seen in a younger age group in PWHV and in those infected with TB (21). With regards to gender as a predisposing factor, some sources state that females are more at risk of developing CADRs than males by 1.5–1.7-fold (32), while other sources say this is only true in the case of antimicrobials and anti-inflammatory drugs (33). Gender was not found to be a significantly predisposing factor in our cohort, with 49.2% of overall cases being female.

Deeply pigmented skin tones remain globally underrepresented in dermatology literature, with potential dire implications in conditions, such as SCAR where early recognition and removal of potentially offending agents may be lifesaving (10). Thus, in this cohort of SCAR in higher Fitzpatrick skin tones, we have highlighted in Figure 1 a series of photographs that demonstrate both typical and atypical features of DRESS and SJS/TEN such as atypical targetoid lesions and purpuric papules that show more subtle erythema and duskiness against deeply pigmented skin. Factors that may influence these differences in cutaneous clinical findings include the quantity and quality of melanin and melanocytes, predilection to scarring and dyspigmentation, as well as propensity to dryness and itching that has been reported in pigmented skin (10).

Immune-mediated adverse drug reactions are drug and phenotype specific, therefore accurate phenotyping is critical for almost any meaningful study aimed at understanding long-term outcomes, offending drug-phenotype natural history and identify clinical predictors or biomarkers. Furthermore, given the poor sensitivity of diagnostic tools to identify culprit drug (34, 35), detailed drug exposure histories may be the only available tool for attributing drug causality. Validation of phenotyping in CADRs and SCAR has thus been a focus of extensive research. RegiSCAR validation system, the best established, and most importantly requires skin photographs collected across the SCAR time-course, with adjunctive histology and laboratory measures improving case certainty (36). The comparison of retrospective and prospective arms of this cohort highlights the difficulties with studying SCARs or CADR using routine data collected without electronic medical records. Up to a quarter of drug causality assessment or phenotyping validation could not be completed from retrospective data. The need to have dedicated resources to ensure sufficient and accurate data is collected for understanding SCAR is true globally (37), but likely will require even greater resources and training in lower- middle income African setting. Our AFRSICAR network has mission to develop the tools and training, and attract the resources necessary, for the study of SCAR against the diverse genomic backgrounds of the African continent.

Our study had several limitations largely related to the retrospective arm where data collection required accessing paper-based hospital folders without skin photographs. Due to the retrospective nature of the study and the variables collected, the data was also insufficient to calculate important prognostic scores such as SCORTEN. Additionally, the two SJS/TEN cases found in the death records that were not enrolled in the IMARI prospective cohort were analysed as part of the retrospective cohort. This could have slightly skewed the data to show more deaths in the retrospective cohort. However, the similarity in overall patterns of CADR and offending drugs between prospective and retrospective arms suggests a consistent epidemiological pattern. This is also a single centre tertiary study, and thus may have referral bias, with milder CADR or even certain SCARs being managed at lower-level non-dermatology specialist hospitals.

In summary, this study describes an important cohort of vulnerable patients, and several unique aspects of CADR in the context of African HIV and TB endemic settings. SCAR remains a resource intensive, life-threatening adverse event most commonly in PWH and linked to the use of either prophylactic cotrimoxazole or first-line anti-TB drugs. The National Institutes of Health and European and Developing Countries Clinical Trials Partnership-funded IMARI registry and biorepository highlights the benefit of prospective data collection for precision phenotyping CADR and is an invaluable asset for the developing global understanding of CADR given this unique population. Further effort is required to accumulate larger prospective datasets across diverse African settings and increase efforts to identify predictive biomarkers relevant to these vulnerable populations.

## Data Availability

The original contributions presented in the study are included in the article/Supplementary Material, further inquiries can be directed to the corresponding author.
